# Anesthetic Management of Perioperative Extracorporeal Membrane Oxygenation (ECMO) for Thoracoscopic Bullectomy in a Single-Lung Patient: A Case Report

**DOI:** 10.7759/cureus.115165

**Published:** 2026-08-25

**Authors:** Cidália Marques, Inês Graça, Ângela Oliveira, Alexandra Borges, Marta Guerra

**Affiliations:** 1 Anesthesiology, Unidade Local de Saúde do Alto Ave, Guimarães, PRT; 2 Anesthesiology, Unidade Local de Saúde de São João, Porto, PRT; 3 Anesthesiology, Unidade Local de Saude do Alto Ave, Guimarães, PRT

**Keywords:** anesthesia for pneumonectomy, pneumothorax, thoracic anesthesia, veno-venous extracorporeal membrane oxygenation (vv ecmo), video-assisted thoracoscopic surgery

## Abstract

Thoracic surgery in patients with severely limited pulmonary reserve may be unsafe or infeasible with conventional one-lung ventilation. In patients with previous pneumonectomy, surgical exclusion of the only functioning lung abolishes native ventilation, making conventional lung isolation impossible. Venovenous extracorporeal membrane oxygenation (VV-ECMO) may provide temporary respiratory support in carefully selected patients by maintaining oxygenation and carbon dioxide removal while allowing optimal surgical exposure. We report the case of a 79-year-old man with single-lung physiology following left pneumonectomy who presented with recurrent right pneumothorax and underwent uniportal video-assisted thoracoscopic surgery (VATS) bullectomy supported by perioperative VV-ECMO. Peripheral VV-ECMO was established using bilateral femoral venous cannulation, permitting alternating periods of controlled apnea and protective mechanical ventilation while maintaining adequate gas exchange. Following surgery, ECMO support was electively continued during the early postoperative period because of the patient's severely limited respiratory reserve and was successfully weaned 36 hours after surgery. The postoperative course was complicated by bilateral femoral deep venous thrombosis after decannulation, requiring therapeutic anticoagulation. This case illustrates that perioperative VV-ECMO may represent a feasible strategy for carefully selected high-risk thoracic surgical patients in whom conventional lung isolation is not possible. It also highlights the importance of meticulous multidisciplinary planning, individualized anticoagulation strategies, and routine surveillance for cannula-related thrombotic complications.

## Introduction

Thoracic surgery poses significant challenges for the anesthesiologist as it requires maintaining adequate oxygenation and carbon dioxide elimination while ensuring optimal surgical exposure. This is typically achieved through one-lung ventilation (OLV) using a double-lumen endotracheal tube; however, in some patients, OLV may be technically impossible or insufficient, demanding alternative strategies for respiratory support [1].

Extracorporeal membrane oxygenation (ECMO) provides temporary extracorporeal gas exchange and, depending on the configuration, circulatory support. Venovenous ECMO (VV-ECMO) is indicated for isolated respiratory failure, whereas venoarterial ECMO (VA-ECMO) provides both respiratory and hemodynamic support. In patients with preserved cardiac function requiring only respiratory support, VV-ECMO avoids the additional risks associated with arterial cannulation while maintaining adequate oxygenation and carbon dioxide removal [2,3].

Patients with previous pneumonectomy represent a unique physiological challenge. Conventional thoracic surgery relies on temporary exclusion of the operative lung from ventilation to create an adequate surgical field. In a patient with a single remaining lung, however, surgical isolation of that lung would abolish native ventilation, making conventional lung isolation impossible. In this setting, VV-ECMO may provide an alternative means of gas exchange, allowing surgery to proceed without ventilation [1-3].

Although the use of ECMO has expanded the therapeutic options for carefully selected high-risk thoracic surgical patients, its use remains complex and should be balanced against potential complications, including bleeding, thrombosis, vascular injury, infection, hemolysis, and neurological events [1]. Careful patient selection and multidisciplinary planning are therefore essential.

Previous reports have described the use of ECMO to facilitate thoracic procedures in patients with severely limited pulmonary reserve, including selected post-pneumonectomy cases [4-6]. However, reports detailing the perioperative anesthetic management of uniportal video-assisted thoracoscopic surgery (VATS) bullectomy in a patient with single-lung physiology remain scarce. We present the anesthetic and ECMO management of a patient with previous pneumonectomy who underwent uniportal VATS bullectomy supported by perioperative VV-ECMO using an anticoagulation-free strategy. Although the procedure was completed without intraoperative complications and postoperative respiratory recovery was successfully achieved, the patient developed bilateral femoral deep venous thrombosis after decannulation, highlighting that while this approach may be feasible in carefully selected patients, it carries a meaningful risk of cannula-associated thrombotic complications.

## Case presentation

A 79-year-old man (64 kg, 175 cm), fully independent in activities of daily living and enrolled in a senior university, was admitted to the pulmonology ward with recurrent right spontaneous pneumothorax. His medical history was notable for a left pneumonectomy with mediastinal lymph node dissection performed seven years earlier for stage I lung adenocarcinoma, with no evidence of recurrence. He was an ex-smoker (50 pack-years) with chronic obstructive pulmonary disease (COPD), Global Initiative for Chronic Obstructive Lung Disease (GOLD) 2B, without pulmonary hypertension, chronic kidney disease with a solitary right kidney following nephrectomy for clear cell carcinoma, type 2 diabetes mellitus, dyslipidemia, and hypertension. His American Society of Anesthesiologists (ASA) physical status was IV.

**Figure 1:**
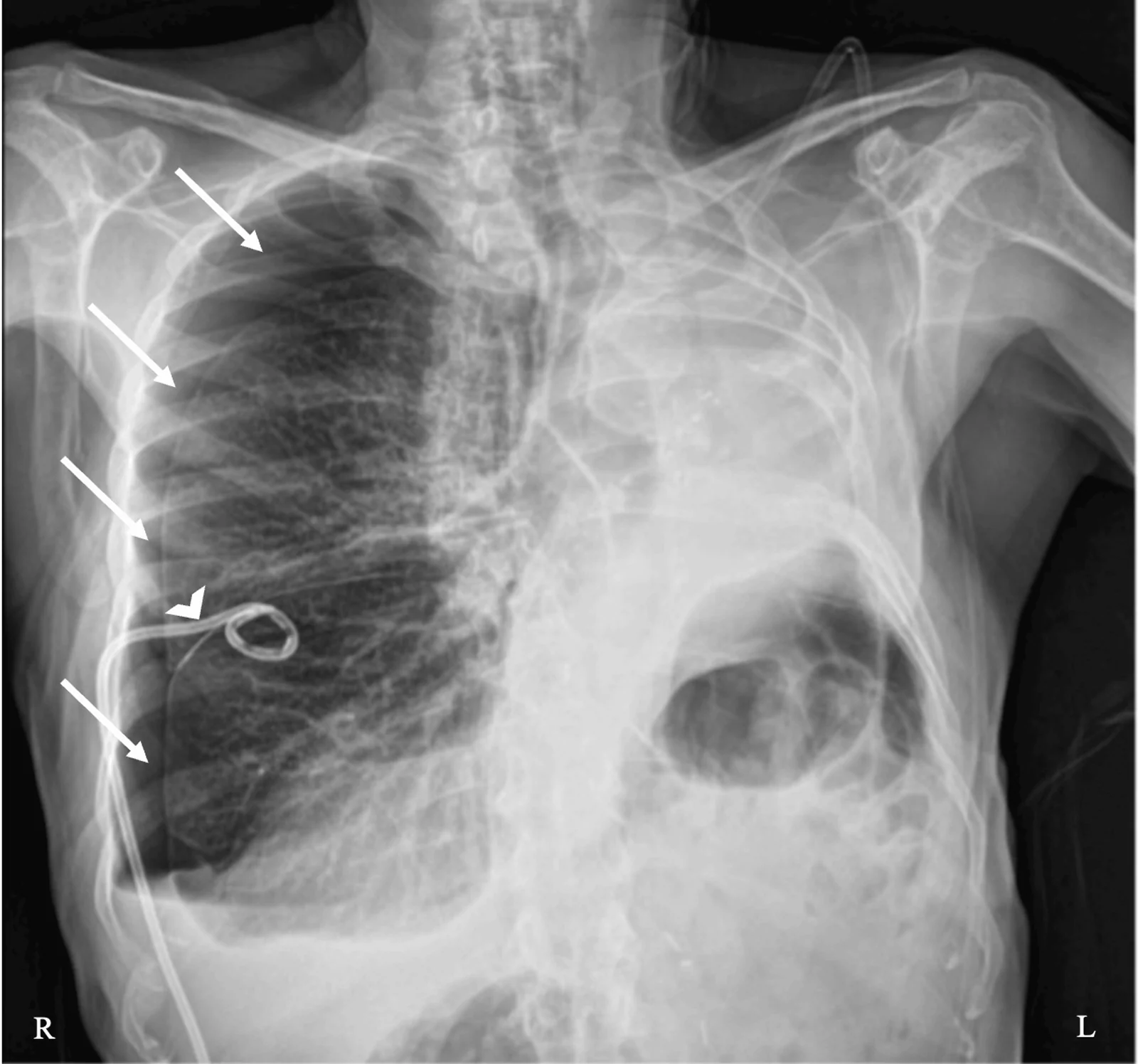
Preoperative chest radiograph Chest radiograph obtained 12 days before surgery, demonstrating recurrent right pneumothorax (arrows), right-sided chest tube (arrowhead), and expected post-pneumonectomy changes (opacification of the left hemithorax, leftward mediastinal shift, and elevation of the left hemidiaphragm). L: left; R: right

During the two months preceding admission, he required two hospitalizations for recurrent right spontaneous pneumothorax. Management consisted of chest tube drainage followed by chemical pleurodesis using talc slurry administered through the chest tube. He presented again to the emergency department with worsening dyspnea and hypoxemia, and a right-sided chest tube was inserted. Subsequently, no respiratory fluctuation (“tiding”) was observed in the water-seal chamber, raising concern for inadequate chest tube function. Chest radiography demonstrated a recurrent right pneumothorax despite the chest tube remaining in situ (Figure 1). Given the persistent pneumothorax and concern for inadequate drainage, a second right-sided chest drain was inserted.

**Figure 2:**
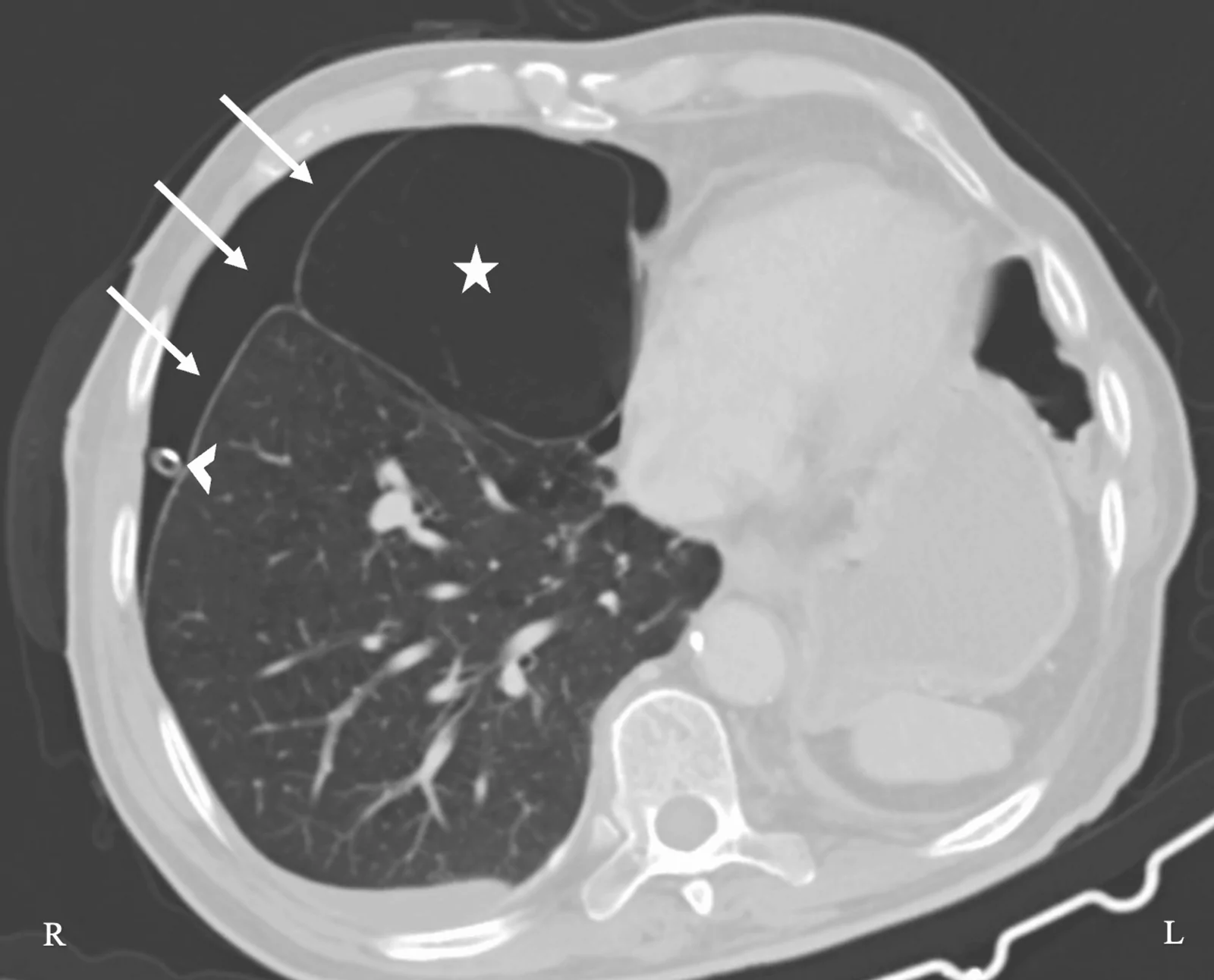
Preoperative chest computed tomography Chest computed tomography performed five days before surgery demonstrating a large mediastinal emphysematous bulla arising from the right middle lobe (star), right pneumothorax (arrows), and thoracic drain in situ (arrowhead). L: left; R: right

A subsequent chest computed tomography (CT) scan demonstrated a mediastinal emphysematous bulla arising from the right middle lobe, persistent right pneumothorax, and the right thoracic drain in situ (Figure 2). Despite adequate drainage and previous talc pleurodesis, a persistent air leak remained, indicating failure of conservative management and prompting consideration of surgical treatment.

Because surgical exclusion of the patient's only functioning lung would abolish native ventilation, conventional lung isolation was impossible. Following multidisciplinary discussion involving thoracic surgeons, intensivists, anesthesiologists, and the ECMO team, the patient was scheduled for VATS bullectomy supported by perioperative VV-ECMO.

The most recent pulmonary function tests, performed one year before surgery, demonstrated a forced expiratory volume in one second (FEV_1_) of 40% predicted and a diffusing capacity for carbon monoxide (DLCO) of 61% predicted. Before surgery, the patient required supplemental oxygen (fraction of inspired oxygen (FiO_2_) of 36%) to maintain a resting oxygen saturation of 96%. Arterial blood gas analysis revealed a pH of 7.36, a PaCO_2_ of 49 mmHg, and a PaO_2_ of 87 mmHg. Blood pressure was 138/71 mmHg, and heart rate was 66 beats/min.

Preoperative laboratory investigations revealed a hemoglobin concentration of 13.0 g/dL, platelet count of 173 × 10^9^/L, serum creatinine of 1.05 mg/dL (estimated glomerular filtration rate 66 mL/min/1.73 m^2^), prothrombin time of 13.6 seconds, activated partial thromboplastin time of 31.5 seconds, and fibrinogen concentration of 394 mg/dL. Doppler ultrasound confirmed patent femoral and internal jugular veins suitable for cannulation.

The patient was admitted to the intensive care unit (ICU) the day before surgery for optimization, where a right radial arterial catheter and a right internal jugular central venous catheter were inserted. On the day of surgery, anesthesia was induced in the ICU with intravenous fentanyl 100 μg, propofol 80 mg, and rocuronium 50 mg, followed by endotracheal intubation with a single-lumen endotracheal tube using direct laryngoscopy. Peripheral VV-ECMO was established using a bilateral femoral venous configuration, with a 23-Fr multistage drainage cannula inserted through the right femoral vein and a 19-Fr return cannula inserted through the left femoral vein. Cannula positions were confirmed by transesophageal echocardiography, with the drainage cannula tip positioned at the inferior vena cava just distal to the junction of the hepatic vein and the return cannula tip within the right atrium, minimizing recirculation. The ECMO circuit was primed with isotonic saline solution before cannulation. Following multidisciplinary assessment balancing the risk of perioperative bleeding against the anticipated short duration of ECMO support, high circuit blood flow, and the absence of pre-existing thromboembolic disease, no heparin bolus was administered during cannulation, and systemic anticoagulation was withheld throughout ECMO support. The patient was subsequently transferred to the operating room accompanied by the ICU and ECMO teams for definitive surgical treatment.

After transfer to the operating room, total intravenous anesthesia (TIVA) was maintained with a continuous propofol infusion titrated according to the Bispectral Index (BIS™; Medtronic, Minneapolis, Minnesota, USA; target 40-60) and fentanyl at 100 μg/h. Neuromuscular blockade was monitored using train-of-four (TOF) stimulation, and a total of 100 mg of rocuronium was administered throughout the procedure.

Mechanical ventilation was maintained using a protective pressure-controlled ventilation (PCV) strategy during periods of native lung ventilation, with tidal volumes ranging from 307 to 343 mL (approximately 5 mL/kg predicted body weight), positive end-expiratory pressure (PEEP) of 5-6 cmH_2_O, peak inspiratory pressure (PIP) between 16 and 20 cmH_2_O, respiratory rate of 12-14 breaths/min, and an estimated driving pressure maintained below 15 cmH_2_O. Fraction of inspired oxygen was adjusted between 40% and 50%.

VV-ECMO blood flow remained stable at approximately 4 L/min throughout the procedure. Sweep gas flow was initially maintained at 1 L/min during periods of mechanical ventilation and increased to 3-4 L/min during planned apneic periods to facilitate carbon dioxide elimination while maintaining physiological acid-base balance. Oxygenator performance remained stable throughout the procedure, with no evidence of circuit thrombosis, oxygenator dysfunction, excessive pressure gradients, or clinically significant recirculation.

To optimize surgical exposure, two planned periods of complete apnea were instituted under full extracorporeal respiratory support. The first apneic period lasted 28 minutes, followed by resumption of protective mechanical ventilation. A second apneic period of 21 minutes was subsequently required to complete surgical dissection. Throughout both apneic periods, extracorporeal gas exchange remained adequate, maintaining satisfactory oxygenation and carbon dioxide removal without hemodynamic instability. No vasopressors or inotropic support were required during the procedure.

Serial arterial blood gas analyses demonstrated adequate respiratory support throughout surgery, with pH remaining between 7.42 and 7.52, PaCO_2_ between 26 and 37 mmHg, and PaO_2_ consistently above 170 mmHg despite prolonged periods of apnea (Table 1). Sweep gas flow was adjusted according to serial blood gas analyses to maintain physiological pH.

**Table 1:** Respiratory support, ventilatory parameters, and arterial blood gas analyses throughout the perioperative course ECMO: extracorporeal membrane oxygenation; FiO_2_: fraction of inspired oxygen; PaCO_2_: partial pressure of arterial carbon dioxide; PaO_2_: partial pressure of arterial oxygen; PEEP: positive end-expiratory pressure; PIP: peak inspiratory pressure; RR: respiratory rate. A dash indicates that the parameter was not applicable.

Time point		ECMO blood flow (L/min)	Sweep gas (L/min)	pH	PaCO_2 _(mmHg)	PaO_2_ (mmHg)	FiO_2_ (%)	Tidal volume (mL)	PIP (cmH_2_O)	PEEP (cmH_2_O)	RR (breaths/min)
Preoperative	Day before surgery (spontaneous breathing)	-	-	7.36	49	87	36	-	-	-	-
	After mechanical ventilation and VV-ECMO initiation (day of the surgery)	4.03	1	7.43	35	176	40	339	16	5	14
Intraoperative	Beginning of surgery (protective ventilation)	3.89	1	7.43	37	247	45	343	16	5	14
	First planned apneic period	4.01	4	7.46	32	210	-	-	-	-	-
	Ventilation resumed	3.97	1	7.42	36	176	50	307	20	6	12
	Second apneic period (early)	4.06	4	7.52	26	278	-	-	-	-	-
	Second apneic period (late)	4.11	3	7.48	31	322	-	-	-	-	-
	End of surgery	3.93	2	7.45	33	246	40	312	19	6	14
Postoperative	ICU admission	3.98	1	7.39	43	161	35	329	19	6	14
	Post-extubation (four hours after surgery)	4.02	1	7.36	41	106	40	-	-	-	-
	Post-decannulation (36 hours after surgery)	-	-	7.35	37	105	28	-	-	-	-
	After chest drain removal (postoperative day six)	-	-	7.38	39	99	24	-	-	-	-
Follow-up	Six-month follow-up	-	-	7.43	42	77	21	-	-	-	-

The total operative time was 90 minutes. A right uniportal VATS bullectomy was successfully performed. Intraoperatively, a large mediastinal emphysematous bulla arising from the right middle lobe was identified and completely resected. Saline air-leak testing performed after reinstitution of ventilation demonstrated no residual air leak. Mechanical pleural abrasion was subsequently combined with thoracoscopic talc poudrage. Although the patient had previously undergone bedside talc slurry pleurodesis, repeat pleurodesis was considered appropriate because definitive resection of the emphysematous bulla addressed the underlying source of the persistent air leak, while direct thoracoscopic talc poudrage combined with pleural abrasion was expected to achieve a more effective and durable pleural symphysis. Intercostal and subpleural infiltration with 0.75% ropivacaine was performed to provide postoperative analgesia. Following meticulous hemostasis, a single apical chest drain was placed.

The patient remained hemodynamically stable throughout the procedure, with no requirement for vasoactive drugs or blood product transfusion. No intraoperative complications related to anesthesia, ECMO support, or the surgical procedure were observed.

The patient was transferred to the ICU while intubated and was extubated four hours later to high-flow nasal oxygen (HFNO) at 40 L/min with a FiO_2_ of 40%. Post-extubation VV-ECMO support was maintained with a blood flow of 4 L/min and a sweep gas flow of 1 L/min, providing adequate gas exchange while the patient resumed spontaneous breathing.

Given the patient's profoundly limited respiratory reserve resulting from previous pneumonectomy and COPD, continuation of VV-ECMO after surgery had been planned preoperatively to facilitate a gradual transition from extracorporeal to native gas exchange while avoiding excessive ventilatory stress during the immediate postoperative period.

Throughout ECMO support, the circuit remained fully functional, with no evidence of oxygenator dysfunction or circuit thrombosis. Circuit performance was assessed by serial monitoring of oxygenator gas exchange, transmembrane pressure gradients, circuit flows, and routine visual inspection for thrombus formation.

Readiness for ECMO weaning was assessed through repeated multidisciplinary evaluation. Sweep gas flow was progressively reduced to zero while maintaining stable spontaneous ventilation. Decannulation was considered appropriate after the patient demonstrated sustained adequate oxygenation and carbon dioxide clearance without extracorporeal gas exchange, stable serial arterial blood gas analyses, minimal oxygen requirements, preserved respiratory mechanics, and hemodynamic stability. VV-ECMO was successfully discontinued, and decannulation was performed approximately 36 hours after surgery.

Routine Doppler ultrasonography performed following decannulation demonstrated bilateral femoral deep venous thrombosis. Therapeutic anticoagulation with enoxaparin 1 mg/kg twice daily was initiated considering the patient's body weight and estimated glomerular filtration rate of 66 mL/min/1.73 m^2^, with anti-factor Xa monitoring. No bleeding complications occurred during anticoagulation therapy.

**Figure 3:**
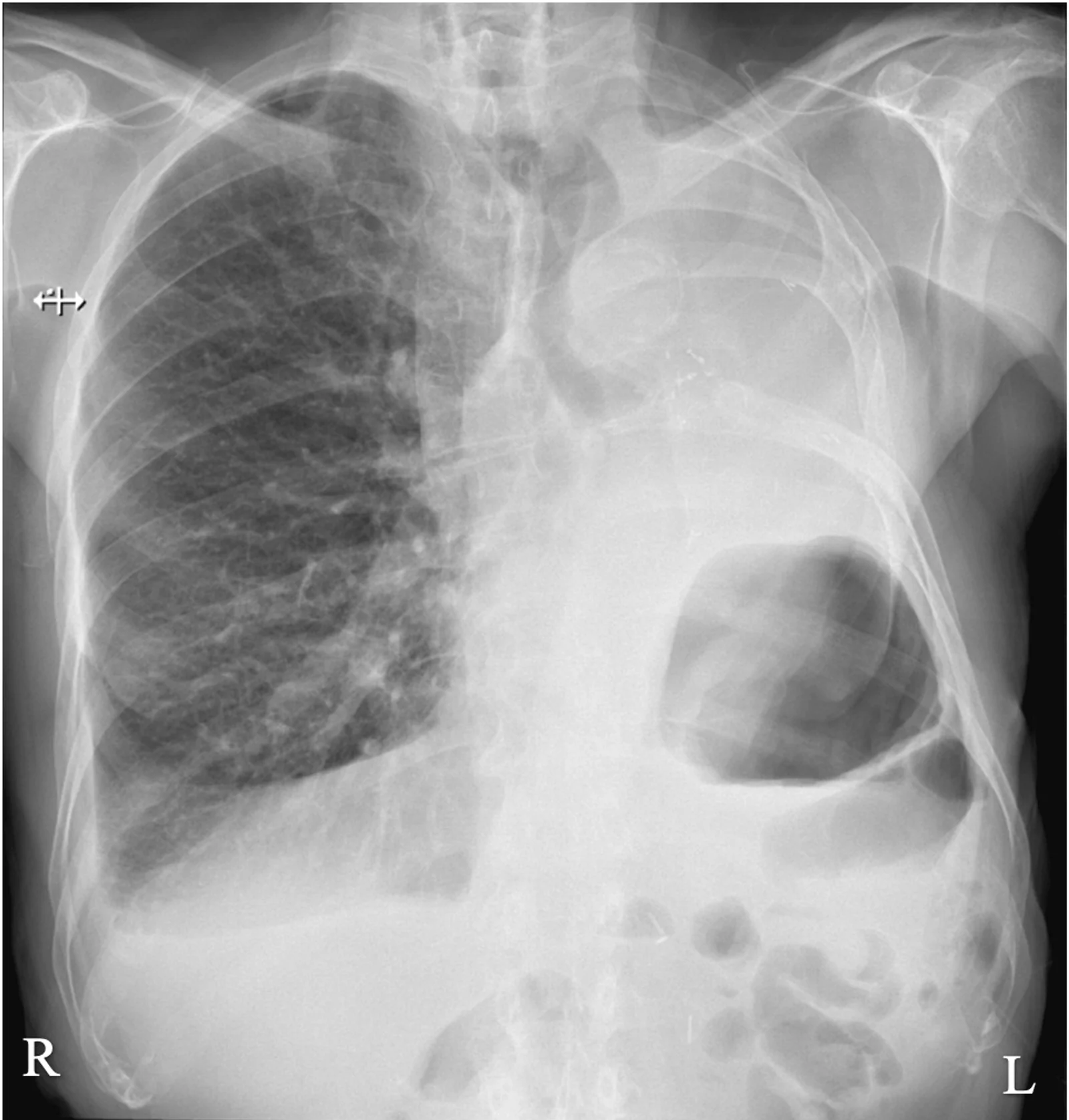
Postoperative chest radiograph after chest drain removal Chest radiograph obtained on postoperative day 6 demonstrating complete re-expansion of the right lung and absence of recurrent pneumothorax. L: left; R: right

The chest drain was removed on postoperative day 6 after complete lung expansion and absence of air leak. Follow-up chest radiography confirmed resolution of the pneumothorax and appropriate expansion of the right lung (Figure 3).

After six days in the ICU, the patient was transferred to the cardiothoracic surgery ward receiving supplemental oxygen (FiO_2_ 24%) with a peripheral oxygen saturation of 96%. He was discharged home on postoperative day 12, breathing room air, with therapeutic anticoagulation planned. A structured respiratory rehabilitation program and multidisciplinary outpatient follow-up were arranged.

At six-month follow-up, the patient remained free of recurrent pneumothorax and had completed six months of therapeutic anticoagulation without further thromboembolic or hemorrhagic complications. He continued respiratory rehabilitation three times weekly. Pulmonary function tests demonstrated a FEV_1_ of 35% predicted and a DLCO of 55% predicted. Peripheral oxygen saturation was 95% on room air, and arterial blood gas analysis showed a pH of 7.43, a PaCO_2_ of 42 mmHg, and a PaO_2_ of 77 mmHg, confirming satisfactory long-term gas exchange despite persistently severe impairment of pulmonary reserve.

A chronological summary of the patient's clinical course is presented in Table 2.

**Table 2:** Clinical timeline from recurrent pneumothorax to six-month follow-up Clinical timeline summarizing the patient's recurrent right pneumothorax, preoperative optimization, VV-ECMO-supported VATS bullectomy, postoperative course, and six-month follow-up. VATS: video-assisted thoracoscopic surgery; VV-ECMO: venovenous extracorporeal membrane oxygenation; FEV_1_: forced expiratory volume in one second; DLCO: diffusing capacity for carbon monoxide; FiO_2_: fraction of inspired oxygen

Time point	Clinical findings, investigations, and management
Seven years before admission	Left pneumonectomy with mediastinal lymph node dissection for stage I lung adenocarcinoma.
Two months before admission	First hospitalization for right pneumothorax treated with tube chest drainage.
One month before admission	Second hospitalization for right pneumothorax requiring chest tube drainage and talc slurry pleurodesis.
Current admission	Recurrent right pneumothorax. Chest CT demonstrated a large mediastinal emphysematous bulla arising from the right middle lobe and persistent pneumothorax, with a persistent air leak despite drainage and previous pleurodesis.
Preoperative evaluation	The patient required supplemental oxygen (FiO_2_ 36%) to maintain a resting oxygen saturation of 96%. Echocardiography demonstrated preserved biventricular function without pulmonary hypertension. Multidisciplinary evaluation led to the decision to perform uniportal VATS bullectomy under perioperative VV-ECMO support.
Day of the surgery	General anesthesia was induced, followed by bilateral femoral VV-ECMO cannulation in the ICU. Uniportal VATS bullectomy, mechanical pleural abrasion, and talc poudrage were subsequently performed. Two planned apneic periods of 28 and 21 minutes were used to optimize surgical exposure. Total operative time was 90 minutes.
Four hours after surgery	The patient was extubated to high-flow nasal oxygen (40 L/min, FiO_2_ 40%). VV-ECMO was maintained because of severely limited respiratory reserve.
36 hours after surgery	VV-ECMO was successfully weaned and decannulated after adequate native gas exchange was demonstrated. Routine Doppler ultrasonography revealed bilateral femoral deep venous thrombosis, and therapeutic enoxaparin was initiated.
Postoperative day 6	The chest drain was removed after complete lung re-expansion and absence of air leak. Chest radiography confirmed complete right lung expansion without recurrent pneumothorax. The patient was transferred from the ICU to the cardiothoracic surgery ward.
Postoperative day 12	The patient was discharged home breathing room air, with therapeutic anticoagulation and respiratory rehabilitation arranged.
Six-month follow-up	No recurrent pneumothorax was observed. The patient had completed six months of therapeutic anticoagulation without bleeding or recurrent thrombosis and continued respiratory rehabilitation. Pulmonary function tests showed FEV_1_ 35% predicted and DLCO 55% predicted.

## Discussion

Thoracic surgery in patients with previous pneumonectomy presents a unique physiological challenge because surgical exclusion of the only functioning lung abolishes native ventilation. In this setting, conventional OLV cannot provide an adequate operative field while maintaining gas exchange. ECMO therefore becomes an attractive adjunct, allowing surgery to proceed while maintaining oxygenation and carbon dioxide removal independently of pulmonary ventilation [1-3].

ECMO can be categorized into VA and VV-ECMO configurations. The ECMO circuit consists of drainage and return cannulas, a pump, a membrane lung, a heat exchanger, an oxygen source, a blender, and connecting tubing [7]. VA-ECMO provides full hemodynamic and respiratory support by draining deoxygenated blood from the venous system and reinfusing oxygenated blood to the arterial system. In contrast, VV-ECMO is indicated when respiratory support alone is required. In VV-ECMO, blood is withdrawn from the venous system, oxygenated via an extracorporeal membrane, and returned to the venous circulation, with systemic oxygen delivery relying on the patient’s native cardiac output [7].

In the present case, VV-ECMO was selected because the patient had preserved biventricular function and required respiratory support alone. Compared with VA-ECMO, VV-ECMO avoids arterial cannulation and its associated complications while providing adequate oxygenation and carbon dioxide removal. This configuration therefore represented the least invasive extracorporeal support capable of meeting the physiological requirements of this procedure [2,4].

Alternative strategies, including non-intubated VATS, high-frequency jet ventilation, and apneic oxygenation with permissive hypercapnia, were considered. However, given the patient's single-lung physiology, COPD, and the anticipated need for prolonged collapse of the operative lung, these techniques were considered unlikely to provide adequate gas exchange or satisfactory surgical exposure. Therefore, perioperative VV-ECMO was selected to provide complete extracorporeal respiratory support while allowing controlled interruption of native ventilation.

The most common cannulation strategies for VV-ECMO include two-site and dual-lumen configurations. In the two-site approach, blood is drained from a femoral vein and returned via the right internal jugular vein. An alternative involves bilateral femoral cannulation, with drainage from one femoral vein and reinfusion through the contralateral vein. Dual-lumen cannulas are inserted via the internal jugular vein, draining blood from both the superior and inferior vena cava and directing reinfusion toward the tricuspid valve, which optimizes venous drainage and reduces recirculation [7]. The choice of bilateral femoral venous cannulation was dictated by procedural considerations. Avoiding internal jugular cannulation facilitated airway management, unrestricted positioning of the patient for right-sided uniportal VATS, and simplified surgical access.

Protective ventilation remains a cornerstone of intraoperative VV-ECMO management. Current recommendations advocate tidal volumes ≤6 mL/kg predicted body weight, low airway pressures, individualized PEEP, and low driving pressures (≤15 mmHg) to minimize ventilator-induced lung injury while preserving alveolar recruitment [8]. Arterial pH should generally be maintained between 7.35 and 7.45 with gradual correction achieved through adjustments in sweep gas flow. Oxygenation depends predominantly on ECMO blood flow, whereas carbon dioxide clearance is primarily sweep-dependent. Blood flow should be maintained at the lowest level that ensures adequate systemic oxygen delivery while minimizing hemolysis and shear-related complications [8].

Our management closely reflected these principles. During periods of native ventilation, PCV achieved tidal volumes of 307-343 mL (4.8-5.4 mL/kg predicted body weight), with PIPs of 16-20 cmH_2_O, PEEP of 5-6 cmH_2_O, and an estimated driving pressure below 15 cmH_2_O. To optimize surgical exposure, ventilation was alternated with two planned apneic periods lasting 28 and 21 minutes. Throughout the procedure, ECMO blood flow remained stable at approximately 4 L/min. Sweep gas flow was maintained at 1 L/min during mechanical ventilation and increased to 3-4 L/min during apnea to compensate for the absence of native carbon dioxide elimination. Serial arterial blood gas analyses confirmed effective extracorporeal gas exchange, with PaCO_2_ ranging from 26 to 37 mmHg and PaO_2_ consistently exceeding 170 mmHg despite prolonged apnea. During the second apneic period, transient respiratory alkalosis developed (pH 7.52, PaCO_2_ 26 mmHg), exceeding the target pH range. This was promptly corrected by reducing the sweep gas flow, restoring physiological acid-base balance. This case highlights the importance of continuous arterial blood gas monitoring and dynamic sweep gas adjustment to achieve individualized carbon dioxide control.

Although volatile anesthetics may be used in selected ECMO-supported procedures, TIVA was chosen because prolonged periods of apnea and minimal ventilation could make delivery of inhalational agents less predictable. Furthermore, the pharmacokinetics of anesthetic drugs may be altered during ECMO owing to changes in circulating blood volume, circuit adsorption, organ dysfunction, circuit characteristics, and duration of support. Consequently, anesthetic management should rely on individualized titration using processed electroencephalographic monitoring and neuromuscular monitoring rather than fixed dosing strategies [5].

An important aspect of this case was the decision to continue VV-ECMO after surgery. This strategy had been planned before surgery because the patient's single-lung physiology, COPD, and markedly limited respiratory reserve were considered to place him at high risk of immediate postoperative respiratory failure. Elective continuation of extracorporeal support allowed extubation four hours after surgery while maintaining adequate gas exchange during spontaneous breathing. Progressive reduction of sweep gas flow enabled staged recovery of native pulmonary function before successful decannulation approximately 36 hours after surgery.

Perhaps the most instructive finding of this report concerns the anticoagulation strategy. Because of the anticipated bleeding risk associated with thoracic surgery, the short planned ECMO duration, peripheral VV configuration, high circuit blood flow, and absence of previous thromboembolic disease, systemic anticoagulation was withheld, and no heparin bolus was administered during cannulation. The ECMO circuit remained free of thrombosis throughout support, but routine Doppler ultrasonography performed after decannulation demonstrated bilateral femoral deep venous thrombosis. Therapeutic anticoagulation was initiated promptly, and the patient completed six months of treatment without bleeding or recurrent thromboembolic events.

This complication represents an important clinical message. Recent reports have shown that heparin-free or low-anticoagulation VV-ECMO may be feasible in carefully selected surgical patients; however, thrombotic complications remain clinically relevant, and management must be individualized according to bleeding risk, expected ECMO duration, circuit characteristics, and patient-specific factors [1,3,5]. Anticoagulation-free VV-ECMO should not be regarded as a universally safe strategy but rather a carefully balanced decision requiring close surveillance. Routine post-decannulation vascular ultrasound may facilitate early detection of cannula-associated thrombosis.

Previous reports describing ECMO-assisted thoracic surgery after pneumonectomy remain limited [4-6]. Compared with previously published cases, this report provides detailed perioperative anesthetic management of uniportal VATS bullectomy using bilateral femoral VV-ECMO, planned alternating apnea and protective ventilation, elective postoperative continuation of ECMO, and an anticoagulation-free strategy. Equally important, it documents the occurrence of bilateral femoral deep venous thrombosis despite an otherwise favorable surgical outcome, highlighting the need to balance bleeding avoidance against thrombotic risk when selecting perioperative anticoagulation strategies.

## Conclusions

This case demonstrates that perioperative VV-ECMO can make uniportal video-assisted thoracoscopic bullectomy feasible in carefully selected patients with single-lung physiology in whom conventional lung isolation is impossible. Bilateral femoral VV-ECMO provided effective extracorporeal gas exchange, allowing alternating periods of protective mechanical ventilation and prolonged controlled apnea while maintaining adequate oxygenation and carbon dioxide elimination. Elective continuation of ECMO during the early postoperative period facilitated staged respiratory recovery before successful decannulation.

However, this case also illustrates that an anticoagulation-free VV-ECMO strategy, although feasible in selected surgical patients to reduce perioperative bleeding risk, carries a meaningful risk of cannula-associated thrombotic complications. The occurrence of bilateral femoral deep venous thrombosis after decannulation underscores the need to carefully balance bleeding and thrombotic risks for each patient and the importance of individualized anticoagulation strategies, routine vascular surveillance, and coordinated multidisciplinary postoperative management.
